# Human Cystic Echinococcosis in West Azerbaijan, Northwest Iran: A Retrospective Hospital Based Survey from 2000 To 2009

**Published:** 2013

**Authors:** H Mohammadzadeh Hajipirloo, A Bozorgomid, T Alinia, KH Hazrati Tappeh, R Mahmodlou

**Affiliations:** 1 Department of Medical Parasitology, School of Medicine, Urmia University of Medical Sciences, Urmia, Iran; 2 Department of Medical Parasitology and Mycology, School of Public Health, Tehran University of Medical Sciences, Tehran, Iran; 3 Department of Epidemiology, School of Medicine, Urmia University of Medical Sciences, Urmia, Iran; 4 Department of General Surgery, School of Medicine, Urmia University of Medical Sciences, Urmia, Iran

**Keywords:** Hydatid cyst, Epidemiology, Iran

## Abstract

**Background:**

The aim of this study was to determine the prevalence of hydatidosis in west Azerbaijan, Iran during a 10 year period (2000-2009).

**Methods:**

We surveyed medical records of infected patients with hydatid cyst who had been operated in four hospitals in Urmia City, the capital of West Azerbaijan Province, Iran. Several parameters were analyzed including age, sex, place of residency, hospitalization time, and the location of cysts.

**Results:**

Of 294 cases, 53.3% were female and 46.7% were male with the mean age of 39.4 years (5–93). The average number of operated cysts per year was 29.4 (0.98/100,000 of population). The most affected age group was 20-30 year olds (18.7% of the cases). Cysts were localized in liver and lung in 57.5% and 21.8% of cases respectively and the average hospitalization time was 9 days. Single organ involvement was seen in the majority of patients and 28 (9.5%) cases had multiple involvement. The distribution of residence in patients showed 108 (36.9%) of them to have urban origin and 185 (63.1%) were rural residents. The lowest number (n = 17) and the highest number of operation (n= 48) recorded in 2000 and 2007, respectively.

**Conclusion:**

The prevalence of hydatidosis is high in this city and further studies are needed for evaluation of economic burden and risk factors for CE in this region.

## Introduction

Human hydatidosis or cystic echinococcosis (CE), is an infection with the larval stage of the tiny dog tapeworm *Echinococcus granulosus*. Dogs are the usual definitive hosts, while human; sheep and other livestock are intermediate hosts (1, 2).

Hydatidosis is a notable public health problem. According to the studies, 2083 new cases were reported in Iran in 2002 to 2007 (3) and the prevalence of CE is estimated 0.61-2/100000 population (4).

Hydatidosis can cause significant morbidity and mortality with considerable economic losses for both of humans and livestock.The overall of economic loss attributable to CE in humans and animals in Iran was annually estimated at about US$232.3 million (95% CI US$103.1–397.8 million) (5). Fasihi stated that, the cost of the disease estimated at 0.03% of the country's gross domestic product (5).

Hydatidosis is endemic in West Azerbaijan Province, besides the human cases, the prevalence in sheep, cattle and water buffalo were respectively 2.7%, 8.6% and 12.9% (6). In addition, the rate of infection with *E. granulosus* in stray dogs of this region is 12.5% (7).

In the present study, we evaluated the rate of hydatidosis infection in 4 hospitals during the 10 yr period in Urmia the center of West Azerbaijan Province in Northwest Iran.

## Materials and Methods

In this retrospective study, after receiving an approval from Ethics Committee of Urmia Medical University, we surveyed medical records of patients referred to four major hospitals in Urmia city (Emam Khomeini, Shaheed Motahhari, Emam Reza and Azerbaijan) during a 10-year period (2000-2009) to find patients who had been diagnosed to suffer from hydatid cyst and gone under surgical operation. People from different parts of the province are mostly referred to these hospitals for surgery. Information including demographic data, number of cysts, organ involvement, morbidity and mortality, relapse and days of hospitalization were collected from medical records of CE patients.

Statistical analysis was carried out by using the SPSS ver. 16 software.

## Results

Overall, 294 cases were operated for CE during the 10 year period in Urmia hospitals. From these cases, 137 patients were male (46.7%) and 156 (53.3%) were female. The average number of operated cysts per year was 29.4 (0.98/100,000 of population).

Their ages were between 5 and 93 years (mean age 39.4).The most affected age group was 20-30 year olds (18.7% of the cases) (Table 1). The distribution of residence in patients showed 108 (36.9%) of them having urban origin and 185 (63.1%) were rural residents (with one missing value).

**Table 1:** Age and sex distribution of surgically confirmed cystic echinococcosis cases

Age group	Number and (%) of cases		
	Male n (%)	Female n (%)	Total n (%)
0-10	8 (5.8)	5 (3.2)	13 (4.4)
10-20	20 (14.6)	29 (18.6)	50 (17)
20-30	24 (17.5)	31 (19.9)	55 (18.7)
30-40	23 (16.8)	23 (14.7)	46 (15.6)
40-50	14 (10.2)	21 (13.5)	35 (11.9)
50-60	15 (10.9)	21 (13.5)	36 (12.2)
60-70	15 (10.9)	14 (9)	29 (9.9)
>70	18 (13.1)	12 (7.7)	30 (10.2)
Total	137 (100)	156 (100)	294 (100)

The highest annually hospitalization and operation rate (48 cases) was seen in 2007 and the lowest (17 cases) in 2000.

Infection in one organ was observed in 266 (90.5%) cases. The liver was the most frequently infected organ (57.5%), followed by the lung (21.8%), kidney (3.1%), spleen and brain (each one in 2.4%). Multi organs affected by hydatid cyst in this study represented 28 (9.5%) and most of them were hepato-pulmonary 9 (3.1%).

Mortality as a surgery complication occurred in five (1.7%) patients. The mean length of hospitalization was 9.3 days. Of 289 cases with recorded cyst numbers, 216 (73.5%) cases had only one cyst and two to five (and more) cysts were seen in 36 (12.2%), 21 (7.1%), 7 (2.4%) and 9 (3.1%) cases, respectively. The number of surgical operations done in 74.1% of patients was just one but 10.2% had two, 2.4 had three, 1.4% had four and 0.6% had more operations.

## Discussion

Echinococcosis is an important disease and a neglected public health problem, especially in rural communities of Iran. The existence of very young children with hydatidosis and the new cases registered every year show that the disease is being actively transmitted in this country.

In the present study, 294 cases of hydatid cyst were operated, the average number of operated cysts per year was 29.4 (0.98/100,000 of population).

Mousavi et al. reported 202 cases in Urmia City during 1991-2001 (10 years) (8). In the present study, from 2000-2005 (6 years) 137 cases reported while from 2006-2009 (4 years), 157 cases were reported. This finding shows that the number of hydatid cyst surgeries in this province has increased in recent years. Although, this increase could be the result of many factors such as improving in documentation of referred cases or new diagnostic methods. However, the actual incidence of CE can be higher as many infections are asymptomatic or do not require surgery and some of the patients never seek treatment. The use of seroepidemiological or radiological methods could be effective for determination of status of hydatidosis in this region.

The average prevalence of hydatidosis in humans has been reported in Hamadan, Kermanshah and Kashan with 1.33, 1.41 and 3/100000/inhabitants and throughout Iran 0.61.2/100,000 (11–14)

In the present study, 53.3% of patients were female. This rate has been reported as 55.7% in Kermanshah (10), 55.9% in Hamedan (9), 57.5% in East Azerbaijan (12) and 49.2% in Turkey (13).

Genetic differences between two gender, and culinary behaviors like tasting potentially infected raw vegetables or food staff before cooking them, can be responsible for this difference. Other probable reasons can be anticipated as females’ desire to geophagy during pregnancy (14) or seeking more medical advices than males (15).

The highest infection rate was observed among the 20-30 years age group. Rokni (2009) stated that cystic echinococosis grows very slowly and so it can be postulated that the original infection might be happened in childhood (14). The most cases of hydatid cyst have been reported among the age groups of 20-40 years in Iran (14).

Our study showed that 63.1% patients were from rural areas and 36.9% from urban areas, perhaps because of the characteristics of its transmission cycle, which involves domestic herbivorous animals (sheep, cattle, etc.) and dogs. The most affected organ was liver (57.5%).The higher rate of hepatic infection is attributed to the fact that liver acts as primary filter in the human body.

In this study, postoperative complications occurred in 6 patients (2%) and the mortality rate was 1.7%. Nourjah et al. and Gulsun et al. reported similar low mortality rates in their studies as well (16, 13).

In our study, the mean length of hospitalization was 9.3 days, and it was much lower than reports of Nourjah et al.(16) and Dopchiz et al. (17) from Iran and Buenos Aires (37 and 11 days respectively).

## Conclusion

The rate of infection with hydatid cyst is high in West Azerbaijan, so serious implementation of control and prevention programs is recommended.
